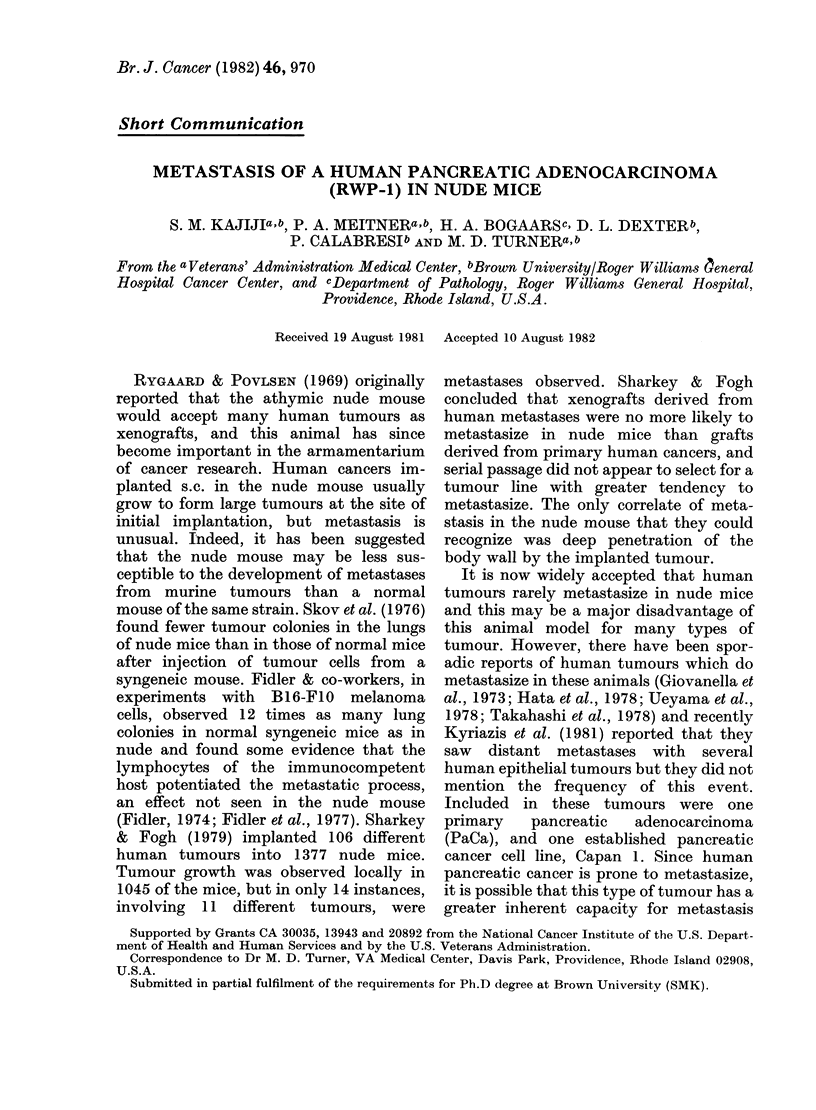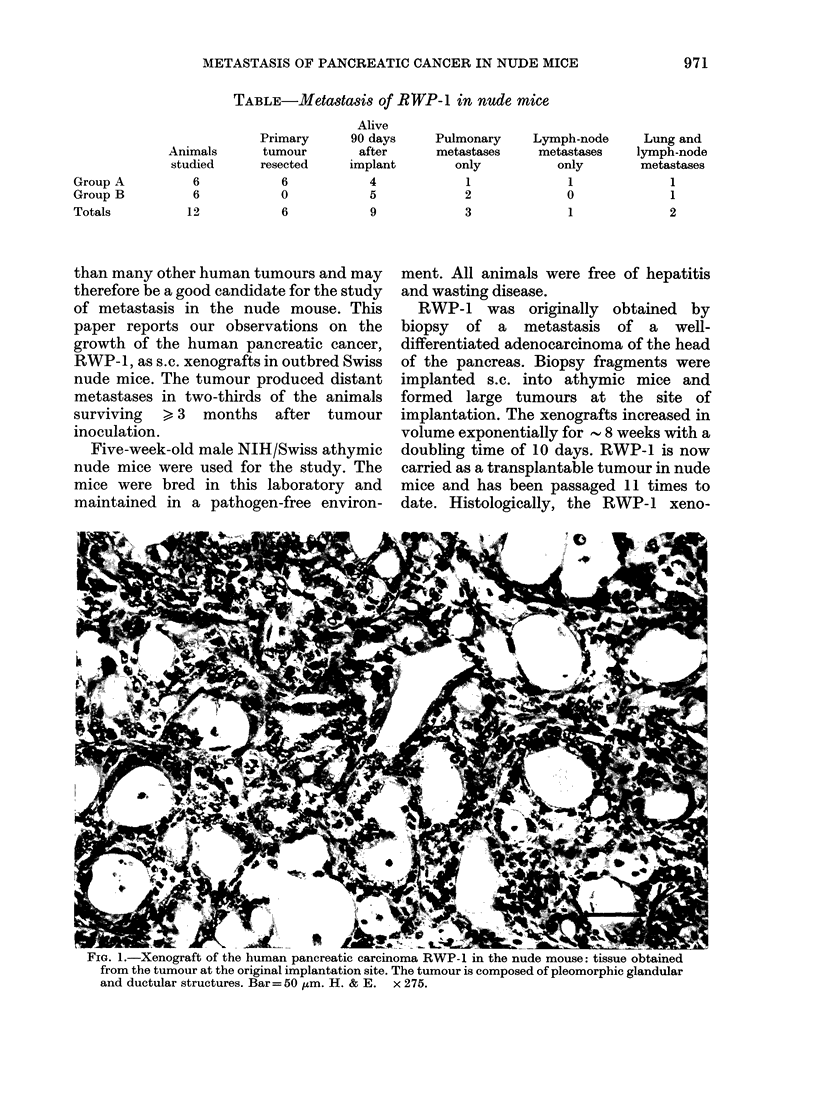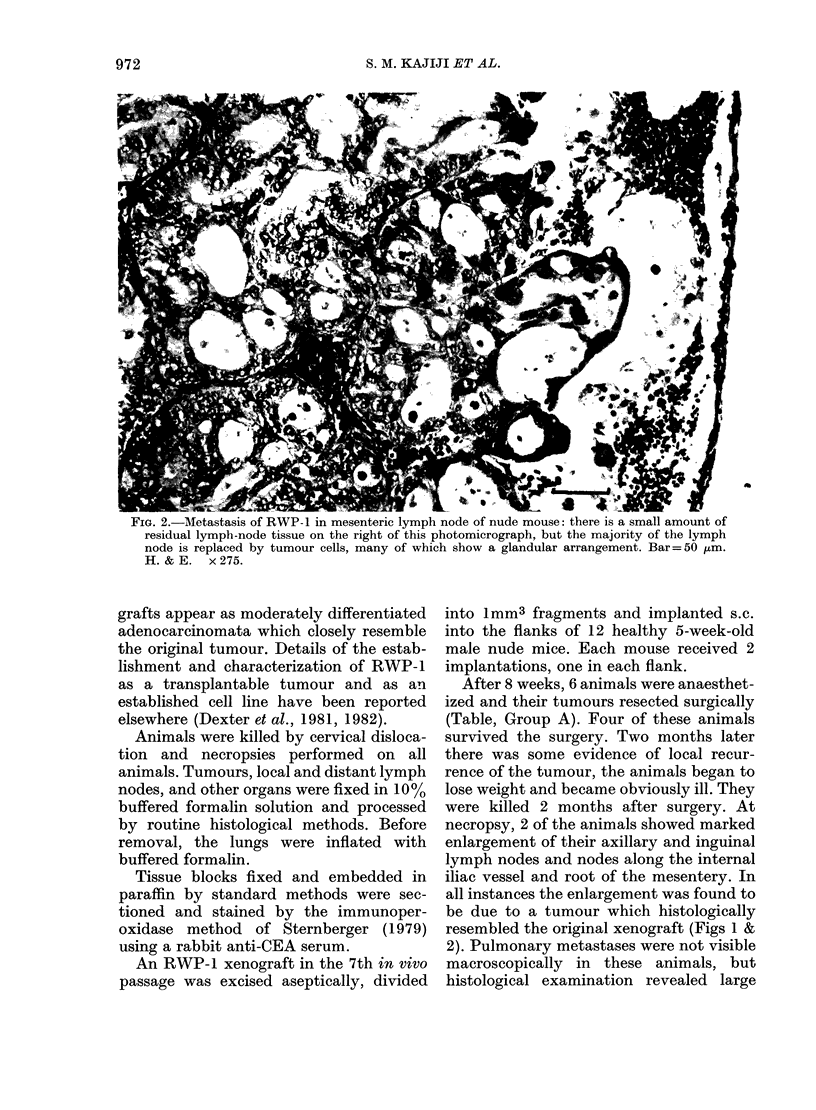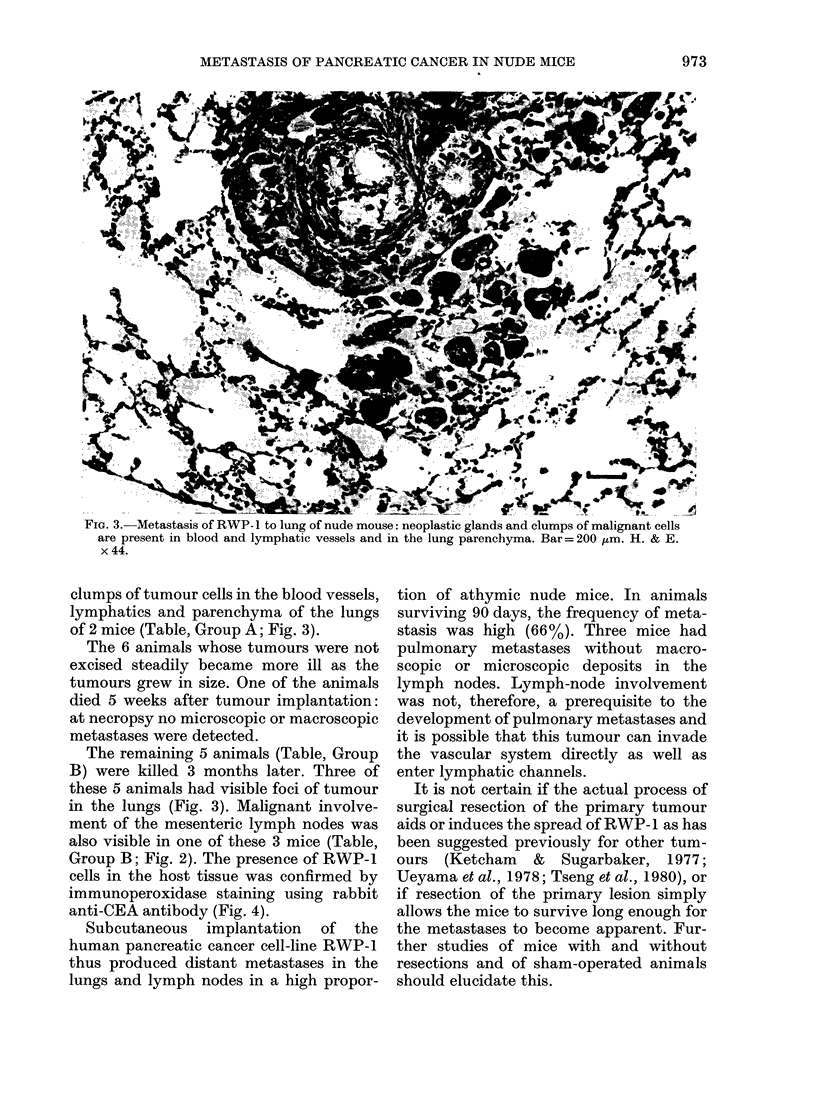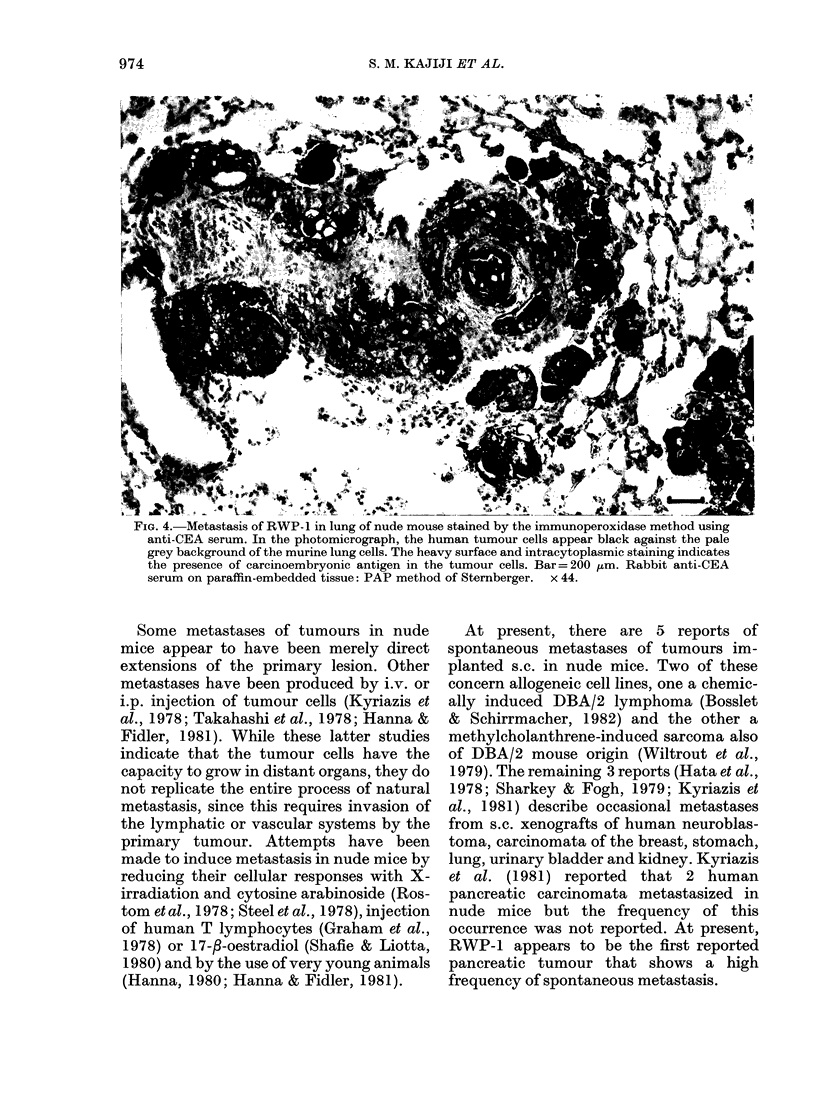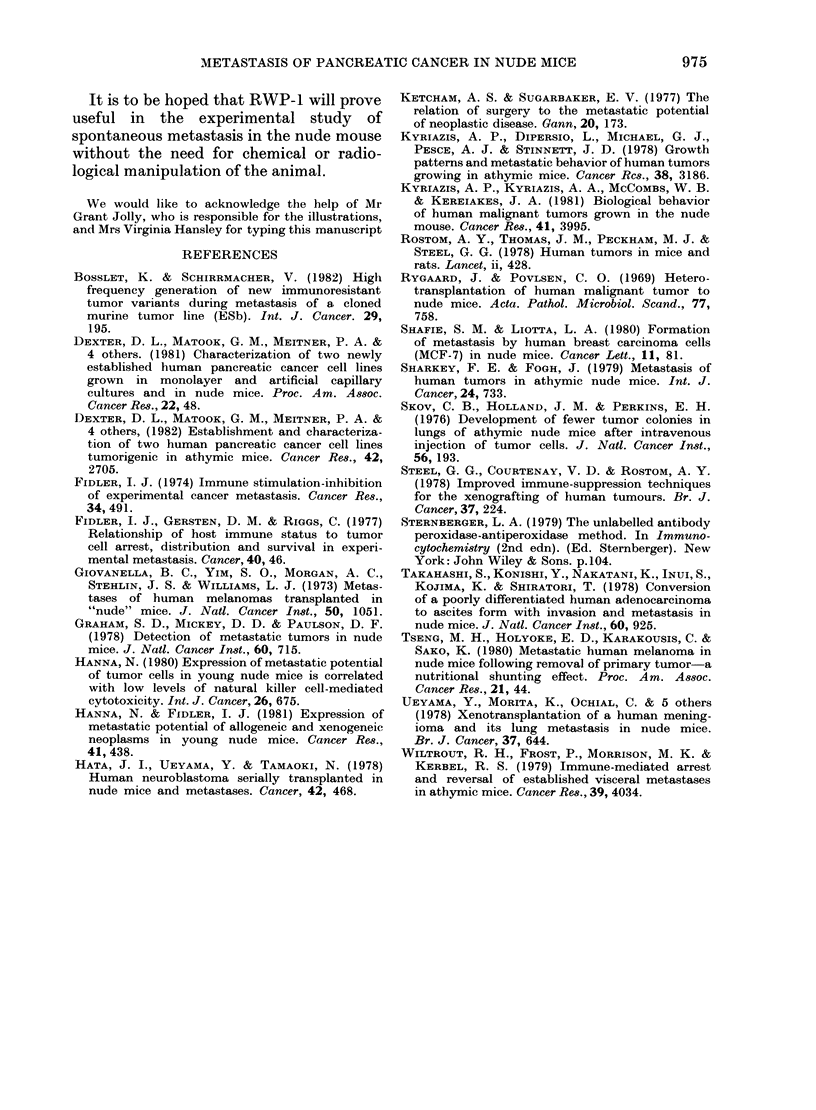# Metastasis of a human pancreatic adenocarcinoma (RWP-1) in nude mice.

**DOI:** 10.1038/bjc.1982.310

**Published:** 1982-12

**Authors:** S. M. Kajiji, P. A. Meitner, H. A. Bogaars, D. L. Dexter, P. Calabresi, M. D. Turner

## Abstract

**Images:**


Br. J. Cancer (1982) 46, 970

*Short Communication*

### METASTASIS OF A HUMAN PANCREATIC ADENOCARCINOMA (RWP-1) IN NUDE MICE

S. M. KAJIJI[a,b], P. A. MEITNER[a,b], H. A. BOGAARS[c], D. L. DEXTER[b], P. CALABRESI[b] AND M. D. TURNER[a,b]

*From the [a]Veterans' Administration Medical Center, [b]Brown University/Roger Williams General Hospital Cancer Center, and [c]Department of Pathology, Roger Williams General Hospital, Providence, Rhode Island, U.S.A.*

Received 19 August 1981 Accepted 10 August 1982

Rygaard & Povlsen (1969) originally reported that the athymic nude mouse would accept many human tumours as xenografts, and this animal has since become important in the armamentarium of cancer research. Human cancers implanted s.c. in the nude mouse usually grow to form large tumours at the site of initial implantation, but metastasis is unusual. Indeed, it has been suggested that the nude mouse may be less susceptible to the development of metastases from murine tumours than a normal mouse of the same strain. Skov *et al.* (1976) found fewer tumour colonies in the lungs of nude mice than in those of normal mice after injection of tumour cells from a syngeneic mouse. Fidler & co-workers, in experiments with B16-F10 melanoma cells, observed 12 times as many lung colonies in normal syngeneic mice as in nude and found some evidence that the lymphocytes of the immunocompetent host potentiated the metastatic process, an effect not seen in the nude mouse (Fidler, 1974; Fidler *et al.*, 1977). Sharkey & Fogh (1979) implanted 106 different human tumours into 1377 nude mice. Tumour growth was observed locally in 1045 of the mice, but in only 14 instances, involving 11 different tumours, were metastases observed. Sharkey & Fogh concluded that xenografts derived from human metastases were no more likely to metastasize in nude mice than grafts derived from primary human cancers, and serial passage did not appear to select for a tumour line with greater tendency to metastasize. The only correlate of metastasis in the nude mouse that they could recognize was deep penetration of the body wall by the implanted tumour.

It is now widely accepted that human tumours rarely metastasize in nude mice and this may be a major disadvantage of this animal model for many types of tumour. However, there have been sporadic reports of human tumours which do metastasize in these animals (Giovanella *et al.*, 1973; Hata *et al.*, 1978; Ueyama *et al.*, 1978; Takahashi *et al.*, 1978) and recently Kyriazis *et al.* (1981) reported that they saw distant metastases with several human epithelial tumours but they did not mention the frequency of this event. Included in these tumours were one primary pancreatic adenocarcinoma (PaCa), and one established pancreatic cancer cell line, Capan 1. Since human pancreatic cancer is prone to metastasize, it is possible that this type of tumour has a greater inherent capacity for metastasis

Supported by Grants CA 30035, 13943 and 20892 from the National Cancer Institute of the U.S. Department of Health and Human Services and by the U.S. Veterans Administration.

Correspondence to Dr M. D. Turner, VA Medical Center, Davis Park, Providence, Rhode Island 02908, U.S.A.

Submitted in partial fulfilment of the requirements for Ph.D degree at Brown University (SMK).

TABLE—*Metastasis of RWP-*1 *in nude mice*

| | Animals studied | Primary tumour resected | Alive 90 days after implant | Pulmonary metastases only | Lymph-node metastases only | Lung and lymph-node metastases |
|---|---|---|---|---|---|---|
| Group A | 6 | 6 | 4 | 1 | 1 | 1 |
| Group B | 6 | 0 | 5 | 2 | 0 | 1 |
| Totals | 12 | 6 | 9 | 3 | 1 | 2 |

than many other human tumours and may therefore be a good candidate for the study of metastasis in the nude mouse. This paper reports our observations on the growth of the human pancreatic cancer, RWP-1, as s.c. xenografts in outbred Swiss nude mice. The tumour produced distant metastases in two-thirds of the animals surviving $\geqslant 3$ months after tumour inoculation.

Five-week-old male NIH/Swiss athymic nude mice were used for the study. The mice were bred in this laboratory and maintained in a pathogen-free environment. All animals were free of hepatitis and wasting disease.

RWP-1 was originally obtained by biopsy of a metastasis of a well-differentiated adenocarcinoma of the head of the pancreas. Biopsy fragments were implanted s.c. into athymic mice and formed large tumours at the site of implantation. The xenografts increased in volume exponentially for $\sim 8$ weeks with a doubling time of 10 days. RWP-1 is now carried as a transplantable tumour in nude mice and has been passaged 11 times to date. Histologically, the RWP-1 xeno-

FIG. 1.—Xenograft of the human pancreatic carcinoma RWP-1 in the nude mouse: tissue obtained from the tumour at the original implantation site. The tumour is composed of pleomorphic glandular and ductular structures. Bar = 50 $\mu$m. H. & E. $\times 275$.

FIG. 2.—Metastasis of RWP-1 in mesenteric lymph node of nude mouse: there is a small amount of residual lymph-node tissue on the right of this photomicrograph, but the majority of the lymph node is replaced by tumour cells, many of which show a glandular arrangement. Bar = 50 μm. H. & E. ×275.

grafts appear as moderately differentiated adenocarcinomata which closely resemble the original tumour. Details of the establishment and characterization of RWP-1 as a transplantable tumour and as an established cell line have been reported elsewhere (Dexter *et al.*, 1981, 1982).

Animals were killed by cervical dislocation and necropsies performed on all animals. Tumours, local and distant lymph nodes, and other organs were fixed in 10% buffered formalin solution and processed by routine histological methods. Before removal, the lungs were inflated with buffered formalin.

Tissue blocks fixed and embedded in paraffin by standard methods were sectioned and stained by the immunoperoxidase method of Sternberger (1979) using a rabbit anti-CEA serum.

An RWP-1 xenograft in the 7th *in vivo* passage was excised aseptically, divided into 1mm$^3$ fragments and implanted s.c. into the flanks of 12 healthy 5-week-old male nude mice. Each mouse received 2 implantations, one in each flank.

After 8 weeks, 6 animals were anaesthetized and their tumours resected surgically (Table, Group A). Four of these animals survived the surgery. Two months later there was some evidence of local recurrence of the tumour, the animals began to lose weight and became obviously ill. They were killed 2 months after surgery. At necropsy, 2 of the animals showed marked enlargement of their axillary and inguinal lymph nodes and nodes along the internal iliac vessel and root of the mesentery. In all instances the enlargement was found to be due to a tumour which histologically resembled the original xenograft (Figs 1 & 2). Pulmonary metastases were not visible macroscopically in these animals, but histological examination revealed large

FIG. 3.—Metastasis of RWP-1 to lung of nude mouse: neoplastic glands and clumps of malignant cells are present in blood and lymphatic vessels and in the lung parenchyma. Bar = 200 μm. H. & E. × 44.

clumps of tumour cells in the blood vessels, lymphatics and parenchyma of the lungs of 2 mice (Table, Group A; Fig. 3).

The 6 animals whose tumours were not excised steadily became more ill as the tumours grew in size. One of the animals died 5 weeks after tumour implantation: at necropsy no microscopic or macroscopic metastases were detected.

The remaining 5 animals (Table, Group B) were killed 3 months later. Three of these 5 animals had visible foci of tumour in the lungs (Fig. 3). Malignant involvement of the mesenteric lymph nodes was also visible in one of these 3 mice (Table, Group B; Fig. 2). The presence of RWP-1 cells in the host tissue was confirmed by immunoperoxidase staining using rabbit anti-CEA antibody (Fig. 4).

Subcutaneous implantation of the human pancreatic cancer cell-line RWP-1 thus produced distant metastases in the lungs and lymph nodes in a high proportion of athymic nude mice. In animals surviving 90 days, the frequency of metastasis was high (66%). Three mice had pulmonary metastases without macroscopic or microscopic deposits in the lymph nodes. Lymph-node involvement was not, therefore, a prerequisite to the development of pulmonary metastases and it is possible that this tumour can invade the vascular system directly as well as enter lymphatic channels.

It is not certain if the actual process of surgical resection of the primary tumour aids or induces the spread of RWP-1 as has been suggested previously for other tumours (Ketcham & Sugarbaker, 1977; Ueyama *et al.*, 1978; Tseng *et al.*, 1980), or if resection of the primary lesion simply allows the mice to survive long enough for the metastases to become apparent. Further studies of mice with and without resections and of sham-operated animals should elucidate this.

FIG. 4.—Metastasis of RWP-1 in lung of nude mouse stained by the immunoperoxidase method using anti-CEA serum. In the photomicrograph, the human tumour cells appear black against the pale grey background of the murine lung cells. The heavy surface and intracytoplasmic staining indicates the presence of carcinoembryonic antigen in the tumour cells. Bar = 200 μm. Rabbit anti-CEA serum on paraffin-embedded tissue: PAP method of Sternberger. × 44.

Some metastases of tumours in nude mice appear to have been merely direct extensions of the primary lesion. Other metastases have been produced by i.v. or i.p. injection of tumour cells (Kyriazis *et al.*, 1978; Takahashi *et al.*, 1978; Hanna & Fidler, 1981). While these latter studies indicate that the tumour cells have the capacity to grow in distant organs, they do not replicate the entire process of natural metastasis, since this requires invasion of the lymphatic or vascular systems by the primary tumour. Attempts have been made to induce metastasis in nude mice by reducing their cellular responses with X-irradiation and cytosine arabinoside (Rostom *et al.*, 1978; Steel *et al.*, 1978), injection of human T lymphocytes (Graham *et al.*, 1978) or 17-β-oestradiol (Shafie & Liotta, 1980) and by the use of very young animals (Hanna, 1980; Hanna & Fidler, 1981).

At present, there are 5 reports of spontaneous metastases of tumours implanted s.c. in nude mice. Two of these concern allogeneic cell lines, one a chemically induced DBA/2 lymphoma (Bosslet & Schirrmacher, 1982) and the other a methylcholanthrene-induced sarcoma also of DBA/2 mouse origin (Wiltrout *et al.*, 1979). The remaining 3 reports (Hata *et al.*, 1978; Sharkey & Fogh, 1979; Kyriazis *et al.*, 1981) describe occasional metastases from s.c. xenografts of human neuroblastoma, carcinomata of the breast, stomach, lung, urinary bladder and kidney. Kyriazis *et al.* (1981) reported that 2 human pancreatic carcinomata metastasized in nude mice but the frequency of this occurrence was not reported. At present, RWP-1 appears to be the first reported pancreatic tumour that shows a high frequency of spontaneous metastasis.

It is to be hoped that RWP-1 will prove useful in the experimental study of spontaneous metastasis in the nude mouse without the need for chemical or radiological manipulation of the animal.

We would like to acknowledge the help of Mr Grant Jolly, who is responsible for the illustrations, and Mrs Virginia Hansley for typing this manuscript

### REFERENCES

Bosslet, K. & Schirrmacher, V. (1982) High frequency generation of new immunoresistant tumor variants during metastasis of a cloned murine tumor line (ESb). *Int. J. Cancer.* **29**, 195.

Dexter, D. L., Matook, G. M., Meitner, P. A. & 4 others. (1981) Characterization of two newly established human pancreatic cancer cell lines grown in monolayer and artificial capillary cultures and in nude mice. *Proc. Am. Assoc. Cancer Res.*, **22**, 48.

Dexter, D. L., Matook, G. M., Meitner, P. A. & 4 others, (1982) Establishment and characterization of two human pancreatic cancer cell lines tumorigenic in athymic mice. *Cancer Res.*, **42**, 2705.

Fidler, I. J. (1974) Immune stimulation-inhibition of experimental cancer metastasis. *Cancer Res.*, **34**, 491.

Fidler, I. J., Gersten, D. M. & Riggs, C. (1977) Relationship of host immune status to tumor cell arrest, distribution and survival in experimental metastasis. *Cancer*, **40**, 46.

Giovanella, B. C., Yim, S. O., Morgan, A. C., Stehlin, J. S. & Williams, L. J. (1973) Metastases of human melanomas transplanted in "nude" mice. *J. Natl. Cancer Inst.*, **50**, 1051.

Graham, S. D., Mickey, D. D. & Paulson, D. F. (1978) Detection of metastatic tumors in nude mice. *J. Natl. Cancer Inst.*, **60**, 715.

Hanna, N. (1980) Expression of metastatic potential of tumor cells in young nude mice is correlated with low levels of natural killer cell-mediated cytotoxicity. *Int. J. Cancer*, **26**, 675.

Hanna, N. & Fidler, I. J. (1981) Expression of metastatic potential of allogeneic and xenogeneic neoplasms in young nude mice. *Cancer Res.*, **41**, 438.

Hata, J. I., Ueyama, Y. & Tamaoki, N. (1978) Human neuroblastoma serially transplanted in nude mice and metastases. *Cancer*, **42**, 468.

Ketcham, A. S. & Sugarbaker, E. V. (1977) The relation of surgery to the metastatic potential of neoplastic disease. *Gann*, **20**, 173.

Kyriazis, A. P., DiPersio, L., Michael, G. J., Pesce, A. J. & Stinnett, J. D. (1978) Growth patterns and metastatic behavior of human tumors growing in athymic mice. *Cancer Rcs.*, **38**, 3186.

Kyriazis, A. P., Kyriazis, A. A., McCombs, W. B. & Kereiakes, J. A. (1981) Biological behavior of human malignant tumors grown in the nude mouse. *Cancer Res.*, **41**, 3995.

Rostom, A. Y., Thomas, J. M., Peckham, M. J. & Steel, G. G. (1978) Human tumors in mice and rats. *Lancet*, ii, 428.

Rygaard, J. & Povlsen, C. O. (1969) Heterotransplantation of human malignant tumor to nude mice. *Acta. Pathol. Microbiol. Scand.*, **77**, 758.

Shafie, S. M. & Liotta, L. A. (1980) Formation of metastasis by human breast carcinoma cells (MCF-7) in nude mice. *Cancer Lett.*, **11**, 81.

Sharkey, F. E. & Fogh, J. (1979) Metastasis of human tumors in athymic nude mice. *Int. J. Cancer*, **24**, 733.

Skov, C. B., Holland, J. M. & Perkins, E. H. (1976) Development of fewer tumor colonies in lungs of athymic nude mice after intravenous injection of tumor cells. *J. Natl. Cancer Inst.*, **56**, 193.

Steel, G. G., Courtenay, V. D. & Rostom, A. Y. (1978) Improved immune-suppression techniques for the xenografting of human tumours. *Br. J. Cancer*, **37**, 224.

Sternberger, L. A. (1979) The unlabelled antibody peroxidase-antiperoxidase method. In *Immunocytochemistry* (2nd edn). (Ed. Sternberger). New York: John Wiley & Sons. p.104.

Takahashi, S., Konishi, Y., Nakatani, K., Inui, S., Kojima, K. & Shiratori, T. (1978) Conversion of a poorly differentiated human adenocarcinoma to ascites form with invasion and metastasis in nude mice. *J. Natl. Cancer Inst.*, **60**, 925.

Tseng, M. H., Holyoke, E. D., Karakousis, C. & Sako, K. (1980) Metastatic human melanoma in nude mice following removal of primary tumor—a nutritional shunting effect. *Proc. Am. Assoc. Cancer Res.*, **21**, 44.

Ueyama, Y., Morita, K., Ochial, C. & 5 others (1978) Xenotransplantation of a human meningioma and its lung metastasis in nude mice. *Br. J. Cancer*, **37**, 644.

Wiltrout, R. H., Frost, P., Morrison, M. K. & Kerbel, R. S. (1979) Immune-mediated arrest and reversal of established visceral metastases in athymic mice. *Cancer Res.*, **39**, 4034.